# Development of a Psychometric Test: A Care Risk Scale for Homebound Older People With Dementia

**DOI:** 10.3389/fpsyg.2022.876173

**Published:** 2022-05-13

**Authors:** Xiaoxin Dong, Lingbo Zhao, Xianbo Kong, Ting Xu, Tongda Sun

**Affiliations:** ^1^Ningbo College of Health Sciences, Ningbo, China; ^2^Ningbo Psychiatric Hospital, Ningbo, China; ^3^Ningbo Kangning Hospital, Ningbo, China

**Keywords:** dementia, care risk, assessment, reliability, validity, older people

## Abstract

**Background:**

Homebound older people with dementia (OPWD) face a series of care risks due to disease characteristics, care issues, and the family environment. However, China lacks a quantitative assessment tool for care risk. Thus, we attempted to develop a care risk scale for homebound OPWD.

**Methods:**

A care risk scale, with initially 18 items, was designed based on a systematic literature review, expert consultation, and a pilot study with 20 OPWD. The initial scale was validated among 1,045 homebound OPWD in Ningbo, China from November 1, 2020 to July 30, 2021. After removing three items that lacked discrimination power, the reliability and validity of the remaining 15 items was evaluated. Factor extraction was performed *via* principal axis factoring and Cattell’s scree plot analysis, with the resulting factors then being subjected to a varimax rotation.

**Results:**

The final scale consisted of 15 items assessed on a 5-point Likert scale that loaded on to three different factors, including dementia symptoms (four items), family support (four items), and home environment (seven items). These three factors were found to explain 72.9% of the cumulative variance. The overall Cronbach’s alpha for the final scale was 0.907. The correlation coefficients in the item-to-total analysis ranged from 0.511 to 0.662.

**Conclusion:**

The validation analysis indicated satisfactory reliability and validity of the 15-item scale for assessing care risk of homebound OPWD. This scale can help long-term care professionals and family caregivers identify care risks and help them take targeted measures to enhance safety of care for OPWD.

## Introduction

There are over 55 million people living with dementia worldwide and this number will reaching 78 million in 2030 (Alzheimer’s Disease International, 2021). Dementia not only manifests as the weakening of physical function, but also the gradual loss of neurological functions, such as memory and orientation, accompanied by a series of mental and behavioral symptoms (Gale et al., 2018). Notably, home care remains the dominant care mode internationally for older people with dementia (OPWD). According to statistics released by Alzheimer’s Disease International (2020), more than 70% of OPWD live at home, and also wish to remain there. Specifically, in China, more than 80% of OPWD are cared for at home (Zhao and Wang, 2020).

However, homebound OPWD are subject to several safety issues. For example, it has been reported that approximately 40% of OPWD have gotten lost in some manner (Neubauer et al., 2021), which is largely due to their memory loss and disorientation (Bantry and Montgomery, 2014). The consequences of them getting lost are serious, with severe examples resulting in injury or even death (Woolford et al., 2017). Moreover, according to reports, 30–50% of OPWD have fallen (Dudevich et al., 2018; Zhang et al., 2019). Herein, a high rate of falling is related to a poor self-care ability and overall orientation, which are common among OPWD (Zhang et al., 2019), in addition to the fact that an unreasonable room layout makes their lived environment unsafe (Green, 2018). Furthermore, the forgetfulness associated with dementia and any inadequate care may both lead to increased medication risks (Poland et al., 2014; Green, 2018), with it being reported that more than 20.8% of OPWD have had at least one medication safety incident (Dudevich et al., 2018). The most difficult challenge faced by caregivers is OPWD’s agitation behaviors, which has been observed in as many as 89% of cases. Improper handling of these behaviors can result in self-injury or injury to others. These agitation behaviors of OPWD are not only related to disease factors, but can also be triggered by the negative communication styles of caregivers or by adverse environmental stimuli (Vugt et al., 2004; Kales et al., 2015).

The development of systematic and scientific risk assessment tools is a key means of risk analysis and response. The Alzheimer’s Association Campaign for Quality Care released the “Dementia Care Practice Recommendations for Professionals Working in a Home Setting” (Alzheimer’s Association, 2009), which outlines the fact that a systematic analysis and prediction of care risk factors is an important basis for home care, with them then providing risk assessment and intervention strategies as guidance for home care. Furthermore, to maximize the safety of OPWD at home, the United Kingdom has promulgated the “Risk Guidance for People with Dementia” (UK Government Department of Health, 2010). Additionally, the American National Institute on Aging has specifically developed a home safety checklist for OPWD (National Institute on Aging, 2017), which advises caregivers in the best ways in which to check safety risks. The safety factors covered in these guidelines and checklists mainly include care and environmental factors. Further, Lee et al. (2019) developed a person-centered risk assessment framework to assess and manage risk for dementia patients, with it suggesting that the risk of a patient engaging in a certain activity can be assessed from the perspective of both them and their caregivers. Another study by Green (2018) conducted a detailed analysis of safety implications of possible risk events and factors for homebound OPWD. Additionally, research by Adams (2001) applied a socially constructed approach to define the care risks of OPWD. Finally, McDermott (2010) proposed care risk factors for dementia, as well as an assessment and management process. In China, many scholars have conducted studies regarding the care risks of OPWD. The “Expert Consensus on Long-term Healthcare of Patients with Cognitive Disorders in China,” released by the Cognitive Impairment Branch of the Chinese Geriatrics Association (2016), states that a daily living ability assessment, a living environment layout, and various care factors should be considered when caring for OPWD. Hao et al. (2016) developed a home-based dementia care assessment list using aspects of daily care, including mental behavior, environmental settings, and living arrangements. Further, Zeng et al. (2018) qualitatively described the home safety hazards of OPWD and proposed various intervention measures. However, these studies only provide a description of possible risks. Internationally, there are various scales, such as the Morse Fall Assessment Scale (Morse, 2002) and the Professional Environmental Assessment Scale (Fleming, 2011), that then go on to also assess risk factors; however, these scales are not specific to OPWD and only target one risk factor dimension.

Based on the extant literature, this study developed a care risk scale for homebound OPWD to help long-term care professionals and family caregivers in quantitatively evaluating the care risks of their patients, identifying key risk factors, predicting the possibility of risk occurrence, and which provides a basis for taking targeted measures to enhance the safety and care of OPWD.

## Materials and Methods

### Ethics Statement

The protocol and content of this study were approved by the Research Ethics Committee of Ningbo College of Health Sciences (NBWY-2019-012). All respondents read and signed an informed consent form prior to participating in the study.

### Development of the Care Risk Assessment Scale

The care risk scale for dementia was developed *via* four steps. First, we systematically reviewed the literature on care risk factors for OPWD to identify factors and establish an initial item pool. Second, we organized an expert consultation meeting comprising six experts in the field of long-term care for dementia to evaluate and revise the initial item pool. Further, we grouped items related to the care risks of homebound OPWD into three factors: (1) dementia symptoms, (2) family support, and (3) home environment. For the three factors, a self-report initial scale containing 16 items was formed. Each item was scored on a 5-point Likert scale ranging from “strongly agree” to “strongly disagree” and coded from 0 to 4. The sum of the scores of all the items was the care risk value. The higher the total score, the greater the care risks. Third, we organized a symposium of 10 OPWD who were selected using purposive sampling. These individuals were interviewed face-to-face using the 16-item initial scale. They were invited to fill out the scale and provide feedback on the items. Subsequently, the scale was revised and expanded to 18 items. Fourth, a pilot study was conducted using the 18-item scale on 20 OPWD who were selected using convenience sampling. The pilot study was conducted to ensure that the items of the scale were clear and understandable to all participants, and the time taken to complete the scale was measured. Finally, the content validity was evaluated be the same six experts. These experts were asked to rate the necessity of each item within the scale on a 3-point scale: 1 = not essential, 2 = useful but not essential, 3 = essential.

### Psychometric Test of the Care Risk Assessment Scale

#### Data Collection, Setting, and Population

The psychometric testing of the 18-item scale was conducted between November 1, 2020 and July 30, 2021, in 103 communities in Ningbo city, Zhejiang province. Trained community medical staff conducted face-to-face household interviews with registered OPWD. The inclusion criteria were: (a) over 60 years old, (b) cared for at their home, (c) diagnosed with dementia by specialists, and (d) able to complete questionnaires on their own. Exclusion criteria were: (a) severely impaired cognitive function and (b) impaired communication. In addition to the data of the risk assessment scale, basic demographic data were also collected.

A total of 1,098 OPWD were identified and selected for inclusion in our study’s interviews, with 53 (4.83%) refusing to participate. Finally, data from a total of 1,045 OPWD were collected. Further, to measure test-retest reliability, a random sample of 100 out of the 1,045 OPWD completed the questionnaire again 20 days later.

#### Statistical Analysis

All analyses were performed using SPSS (version 25.0; SPSS Inc., Chicago, IL, United States) and AMOS 21.0. The reliability and validity of the scale were measured using the analyses below.

##### Item Distribution

The frequency distribution of the options of the 18 items in the scale was analyzed, and the items were deleted when the answers showed a skewed distribution. Generally, if the selection rate of any answer for an item exceeds 80%, the item can be considered for deletion (He et al., 2011).

##### Discrimination Coefficient

The total score for each respondent was calculated and sorted. Respondents who were in the top 25% and bottom 25% of the total scores were recorded, and the average score of these two parts for each item was calculated. The difference between the two average scores was the discrimination coefficient for each item. The larger the difference, the larger the discrimination coefficient; a minimum discrimination coefficient value of 0.5 is accepted (Yin et al., 2012).

##### Exploratory Factor Analysis and Confirmatory Factor Analysis

Exploratory factor analysis was used to understand the factor structure of the scale. Factor extraction was performed *via* principal axis factoring. Cattell’s scree plot analysis and an eigenvalue greater than 1 determined the number of factors that we then retained. Factors above the bend or elbow cut-off point were retained for Cattell’s scree plot analysis. The resulting factors were subjected to varimax rotation. Items were retained in each factor when their loading level were more than 0.5. Before performing it, the Kaiser-Meyer-Olkin (KMO) test and Bartlett’s test for sphericity were performed to test for suitability for factor analysis. The minimum recommended value of the KMO is 0.6 (Beavers et al., 2013). Bartlett’s test for sphericity with statistical significance indicates that the data are distributed in a spherical shape and each variable is independent from others, indicating the sample is suitable for factor analysis (Bartlett, 1954). Confirmatory factor analysis was applied to analyze the overall fit of the factor model, with the goodness of fit index (GFI), adjusted GFI (AGFI), root mean square error of approximation (RMSEA), comparative fit index (CFI), and incremental fit index (IFI) all being used to check the fit.

##### Content Validity

Content Validity Ratio (CVR) proposed by Lawshe (1975) was used to assess the content validity: $C\,V\,R=\frac{n\,e-N/2}{N/2}$. In this formula, *ne* refers to the number of panelists that answered “essential” for each item of the questionnaire, with *N* then reflecting the total number of panelists. When the CVR value is greater than 0, it means that more than half of the experts agree on the item being essential.

##### Cronbach’s Alpha

Cronbach’s α was used to assess the internal consistency of the scale (Cronbach, 1951). Cronbach’s α greater than 0.9 indicates satisfactory reliability, between 0.8 and 0.9 indicates acceptable reliability, between 0.7 and 0.8 indicates that certain items should be revised, and below 0.7 indicates that some items should be deleted.

##### Corrected Item-Total Correlation

This was assessed using the correlation coefficient between the scores of each item of the scale and the scores of the remaining items; a minimum value of 0.3 is accepted (Beavers et al., 2013).

##### Test-Retest and Split-Half Reliability

Test-retest reliability was estimated by intraclass correlation coefficient (ICC) between the scores of the 100 OPWD who completed the questionnaire twice. Split-half reliability was assessed by Pearson correlation between the scores of the odd items and the even items.

## Results

Table 1 presents the demographic characteristics of 1,045 OPWD who completed the care risk scale. There were 558 women (53.39%) and 487 men (46.61%). Most respondents were over 70 years old, accounting for 82.69% of the sample. The average time taken to complete the questionnaire was 8–12 min.

**TABLE 1:** Demographic characteristics of the respondents (*n* = 1,045).

Demographic characteristics	*N*	%
**Gender**		
Male	487	46.61
Female	558	53.39
**Age**		
60–69	181	17.31
70–79	356	34.04
≥80	508	48.65
**Education level**		
Never attended school	287	27.50
Primary school	402	38.45
Junior high school	197	18.88
High school or higher	158	15.17
**Marital status**		
Married	474	45.38
Divorced	46	4.43
Widowed	439	42.00
Un-married	86	8.19

The item analysis results showed that the frequency distribution of each option in all items ranged from 1.1 to 58.6%, which did not exceed the 80% standard. There was no option for a significantly skewed distribution. The discrimination coefficient of each item ranged from 0.294 to 2.128, and three items were deleted because they lacked discrimination power.

Exploratory factor analysis was carried out on the remaining 15 items to determine the construct validity of the scale. The KMO was 0.91, while Bartlett’s test for sphericity yielded extremely significant results (χ^2^ = 13,958.572; *p* < 0.0001). The results of the exploratory factor analysis showed that the factor structure of the scale was best represented by three factors (dementia symptoms, family support, home environment), and these three factors accounted for 72.9% of the cumulative variance of the 15 items. Table 2 shows the resulting three factors, their respective items, and factor loadings. The three factors generated by the exploratory factor analysis were consistent with the three factors conceptualized in the design stage, with 15 items showing salient loadings on corresponding factors and no substantial cross-loadings on others. Confirmatory factor analysis was performed on the three-factor model, and resulting in the following values: GFI = 0.934 > 0.9, AGFI = 0.903 > 0.9, RMSEA = 0.065 < 0.08, CFI = 0.967 > 0.9, IFI = 0.967 > 0.9. The CVR of the 15 items ranged from 0.67 to 1.0.

**TABLE 2:** Factor loadings of the care risk scale for dementia.

Items	% Explained variance	Factor loadings		
		
		F1	F2	F3
**Factor1:dementia symptoms**	22.336			
My memory is as good as ever.		**0.899**	0.053	0.084
I have no problem with my ability to perceive and recognize my surroundings (e.g., time, place, people).		**0.936**	0.051	0.140
My judgment and ability to solve daily problems are as good as ever.		**0.946**	0.090	0.128
I have no problems with mobility and my daily life (washing, walking, etc.).		**0.791**	0.135	0.131
**Factor2: family support**				
When I am in trouble, my family can help me satisfactorily.	20.382	0.013	**0.800**	0.245
My family is very supportive in my new activities or development.		0.138	**0.715**	0.263
I am very satisfied with the emotional response of my family to me.		0.117	**0.898**	0.232
I am very satisfied with the way my family spends time with me.		0.083	**0.875**	0.257
**Factor3: home environment**	30.179			
My home environment makes me feel a sense of security.		0.005	0.184	**0.752**
My home environment improves my perception of space and time.		0.140	0.173	**0.825**
My home environment is fully capable of supporting my maintenance of life skills (including mobility, washing, bathing, toileting, eating, etc.).		0.103	0.158	**0.855**
My home environment provides me with a private space to rest or work.		0.108	0.189	**0.751**
My home environment fully supports me in exercising my personal preferences and choices.		0.149	0.204	**0.767**
I am comfortable with the level of environmental stimuli (light, color, sound) and have no stress at all.		0.065	0.248	**0.771**
My current living environment makes me feel connected to the past.		0.190	0.274	**0.703**

*The factor loadings for the items to be retained in each factor are shown in bold.*

Table 3 presents the results of the reliability analysis. Cronbach’s alpha for the scale was 0.907. The Cronbach’s alpha values for the three factors within the scale were 0.947, 0.921, and 0.929, respectively. The split-half reliability for the scale was 0.863. The test-retest reliability was 0.842. The correlation coefficients in the item-to-total analysis ranged from 0.511 to 0.662. Alpha item deleted (ID α) indicated that removing either item did not improve the internal consistency of the scale. All of these indicate a robust reliability of the scale.

**TABLE 3:** Reliability analysis of the care risk scale for dementia.

Items	CITC	ID α	Cronbach’s alpha	Total cronbach’s alpha
**Factor1: dementia symptoms**			0.947	0.907
My memory is as good as ever.	0.511	0.894		
I have no problem with my ability to perceive and recognize my surroundings (e.g., time, place, people).	0.530	0.892		
My judgment and ability to solve daily problems are as good as ever.	0.555	0.892		
I have no problems with mobility and my daily life (washing, walking, etc.)	0.528	0.892		
**Factor2: family support**			0.921	
When I am in trouble, my family can help me satisfactorily.	0.521	0.893		
My family is very supportive in my new activities or development.	0.588	0.890		
I am very satisfied with the emotional response of my family to me.	0.609	0.890		
I am very satisfied with the way my family spends time with me.	0.596	0.890		
**Factor3: home environment**			0.929	
My home environment makes me feel a sense of security.	0.571	0.891		
My home environment improves my perception of space and time.	0.662	0.888		
My home environment is fully capable of supporting my maintenance of life skills (including mobility, washing, bathing, toileting, eating, etc.)	0.651	0.889		
My home environment provides me with a private space to rest or work.	0.617	0.890		
My home environment supports me in exercising personal preferences and choices.	0.655	0.888		
I am comfortable with the level of environmental stimuli (light, color, sound) and have no stress at all.	0.606	0.890		
My current living environment makes me feel connected to the past.	0.661	0.888		

*CITC, Corrected item-total correlation; ID α, alpha item deleted.*

For the sample data, homebound OPWD had a greater care risk (mean = 25.93, *SD* = 11.92). The care risk value of the home environment factor was the highest (mean = 9.66, *SD* = 6.69). The average care risk for dementia symptoms and family support was 9.35 (*SD* = 4.79) and 6.91 (*SD* = 4.41), respectively. Moreover, the care risk values were different among different groups homebound OPWD in terms of their gender (*p* < 0.05), age (*p* < 0.05) and marital status (*p* < 0.0001). OPWD who were female, aged 60–69 years, or who lived without a spouse had a greater overall care risk than other groups (Table 4).

**TABLE 4:** Care risk values for the homebound older people with dementia.

Demographic characteristics	Total questionnaire			Dementia symptoms			Family support			Home environment		
				
	Mean	*SD*	*P*	Mean	*SD*	*P*	Mean	*SD*	*P*	Mean	*SD*	*P*
**Gender**			0.015			0.000			0.904			0.074
Male	25.00	12.11		8.79	4.92		6.94	4.54		9.27	6.63	
Female	26.83	11.49		9.91	4.60		6.90	4.26		10.02	6.61	
**Age**			0.017			0.000			0.000			0.022
60–69	27.74	12.03		8.90	4.75		8.17	4.50		10.67	6.94	
70–79	24.68	12.75		8.53	4.87		7.14	4.77		9.00	6.72	
≥80	26.09	11.17		10.07	4.65		6.29	3.99		9.73	6.50	
**Education level**			0.110			0.019			0.000			0.028
Never attended school	26.97	11.06		9.64	4.33		7.12	4.44		10.21	6.54	
Primary school	25.87	12.32		9.02	4.98		6.99	4.38		9.86	6.57	
Junior high school	25.21	12.02		8.99	5.01		7.35	4.28		8.87	6.43	
High school or higher	24.09	11.47		10.42	4.72		5.21	4.03		8.46	7.14	
**Marital status**			0.000			0.000			0.000			0.000
Married	22.52	12.64		8.43	5.01		5.56	4.28		8.53	6.76	
Divorced	28.54	9.22		8.48	4.69		9.04	3.99		11.02	5.87	
Widowed	28.12	10.73		10.31	4.50		7.27	3.99		10.54	6.55	
Un-married	31.58	9.55		9.82	4.10		11.41	3.67		10.34	6.36	

*SD, Standard deviation.*

## Discussion

This study developed a 15-item scale for assessing the care risks of homebound OPWD and conducted an item analysis and reliability and validity tests for its validation. The three factors generated by our exploratory factor analysis were consistent with the three factors conceptualized in the design stage, with the correlated factor loadings being high for each factor, which met the recommended value of 0.4 for all factor loadings (Waltz et al., 2016). Furthermore, the cumulative variance of 72.9% of the three factors was higher than the accepted criterion of 40% (Brink and Louw, 2012). These results suggest that the scale is valid. The high Cronbach’s alpha for the overall scale and the three factors indicated a satisfactory reliability.

The final scale contained three factors: dementia symptoms, family support, and home environment, which are similar to those reported in other studies. The “Dementia Care Practice Recommendations for Professionals Working in a Home Setting” released by the Alzheimer’s Association Campaign for Quality Care (Alzheimer’s Association, 2009) proposed that family support plays a vital role in home care for OPWD. Moreover, the recommendations noted that an environmental assessment of the home regarding possible hazards was appropriate. Aspects of the home environment, such as good lighting, comfortable room temperatures, and decreased clutter and other distractions, should reduce a person’s confusion and fear and promote comfort and safety. The “Expert Consensus on the Long-term Healthcare of Patients with Cognitive Disorders in China” (Academy of Cognitive Disorders of China (ACDC) et al., 2016) pointed out that in caring for patients with dementia, an assessment of the personal abilities (e.g., daily living ability, judgment, hobbies), care situation (e.g., family lifestyle, health concept, social support), and living environment (e.g., stability, familiarity, multi-sensory stimulation areas) of OPWD was important.

The scale developed in this study focused on OPWD’s perceptions of their family support and their lived-in environment, which is similar to the studies by Green (2018) and Lee et al. (2019). The assessment framework developed by Lee et al. (2019) aimed to assess whether an activity met the underlying psychosocial needs of OPWD, including having them feel unconditionally accepted and close to others, as well as informing emotional bonds with others. Besides, Lee et al. (2019) considered OPWD’s sense of environment safety was also an important aspect to assess the care risk. The safety implications proposed by Green (2018) suggest that the previous habits of OPWD should be considered in their care planning. The results of our study showed that their living environment is a key factor in the care risk assessment for OPWD, with many studies then proposing the importance of the environment in the care risk assessment for OPWD. The “Home Safety Checklist for Alzheimer’s Disease” developed by the American National Institute on Aging (2017) focused on the safety of the patient’s environment and proposed various items that need to be checked, such as the lighting brightness, step height, and whether the floor is anti-skid, in order to alert OPWD and their caregivers to potential hazards. The safety implications proposed by Green (2018) also focused on the impact of the environmental layout on the care risk of OPWD, with them suggesting that the layout of the kitchen and bathroom should both be safe.

Due to their affected memory, orientation, and judgment, OPWD have a reduced ability to analyze and judge risks and are prone to adverse events such as getting lost or falling. According to the World Health Organization (2015), reduced cognition and disorientation are among the main factors affecting the care risk of OPWD. Zhang et al. (2019) considered the fact that cognitive flexibility and judgment could affect their risk of falls through the resulting increased risk-taking behaviors. Additionally, Smith et al. (2015) suggested that memory loss with dementia could induce problems with medication safety. After 1.5 years of follow-up, Burgio et al. (2007) found that cognitive ability was a key factor in the occurrence of agitation behaviors, with OPWD with severe cognitive impairment exhibiting more anxiety symptoms and were more likely to experience agitation than those with only moderate cognitive impairment. Therefore, in the process of caring for OPWD, their cognitive functions, such as their memory, orientation, and judgment, should be assessed, with targeted training for them being carried out to delay the decline of these functions specifically.

Family members’ help and affective responses are also known to affect the emotions of older people. At the same time, whether a family can support the new activities or the development of older people may affect their care risk. Edwards et al. (2016) pointed out that the relationship between OPWD and their caregivers was closely related to the cognition, physical function, and quality of life of the former. When OPWD lack necessary support, their compressive ability and immune function are both affected (Newcomer et al., 1999; Somme et al., 2012), and then they are prone to agitation and harm. Bessey and Walaszek (2019) found that providing psychological support and spiritual comfort is beneficial to relieve the symptoms of OPWD, thereby improving their mobility. Wolf-Ostermann et al. (2016) found that the level of support received by OPWD had a significant impact on their quality of life. If necessary, support is lacking, their anti-stress ability will be affected and they will be more likely to be exposed to risks. Familiar people and things are important for OPWD. However, caregivers may inevitably showcase some negative emotional reactions and insufficient support for OPWD due to stress, mental exhaustion, and a lack of skills (Cheng, 2017; Pudelewicz et al., 2018). Thus, family support for OPWD should be assessed, with necessary skills training, psychological counseling, and respite services being provided for family caregivers.

Environmental safety, space identification, private space setting, and environmental stimulation all have an impact on care risk. Many studies have pointed out that unstable and disordered environments can induce agitation in OPWD (Goodall, 2006; Marquardt et al., 2014; Wang et al., 2020). For example, too many steps and slippery floors can lead to falls. Thus, the design and layout of the living environment are crucial for the care of OPWD. When designing the living environment for OPWD, its overall safety and the identification of the space should be considered, with an assistive device for reminders then being added. Further, private spaces to rest should be designed for OPWD. Frequent changes of living places should be avoided, with items of special significance to OPWD being placed in obvious positions to enhance the memory of the space.

### Limitations and Further Research

The reliability and validity of the scale developed in this study were satisfactory. This scale could be a reliable tool for the care risk assessment of homebound OPWD. However, this study has several limitations. First, similar assessment tools were not used to validate the scale. Second, the study was conducted in one city and may not be representative of OPWD from other areas. Third, the study excluded older people with severe cognitive impairment and who were unable to communicate properly; however, they also face care risks. Future studies should be conducted to further test the applicability and generalizability of the scale.

## Conclusion

This study developed a scale to evaluate the care risks of homebound OPWD. Multiple analyses were run to determine the validity and reliability of this scale. The validation analysis indicated satisfactory reliability and validity of the 15-item scale in assessing care risk for homebound OPWD. The scale will help long-term care professionals and family caregivers identify care risks and help them take targeted measures to improve care safety for OPWD.

## Data Availability Statement

The original contributions presented in the study are included in the article/supplementary material, further inquiries can be directed to the corresponding author/s.

## Ethics Statement

The studies involving human participants were reviewed and approved by the Research Ethics Committee of Ningbo College of Health Sciences. Participants provided their written informed consent to participate in the study.

## Author Contributions

XD conceived, designed the study, analyzed the data, and drafted the manuscript. LZ conducted the study and supported the interpretation of the data. XK and TX contributed to data collection. TS critically revised the manuscript and supported the interpretation of the results. All authors have read and approved the final manuscript.

## Conflict of Interest

The authors declare that the research was conducted in the absence of any commercial or financial relationships that could be construed as a potential conflict of interest.

## Publisher’s Note

All claims expressed in this article are solely those of the authors and do not necessarily represent those of their affiliated organizations, or those of the publisher, the editors and the reviewers. Any product that may be evaluated in this article, or claim that may be made by its manufacturer, is not guaranteed or endorsed by the publisher.

## References

[B1] Academy of Cognitive Disorders of China (ACDC) Han Y. Jia J. Li X. Lv Y. Sun X. (2016). Expert Consensus on the Care and Management of Patients with Cognitive Impairment in China. *Chin. J. Gerontol.* 35 1051–1060. 10.1007/s12264-019-00444-y 31792911 PMC7056776

[B2] Adams T. (2001). The social construction of risk by community psychiatric nurses and family carers for people with dementia. *Health Risk Soc.* 3 307–319. 10.1080/13698570120079903

[B3] Alzheimer’s Association (2009). *Dementia Care Practice Recommendations for Professionals Working in a Home Setting.* Chicago: Alzheimer’s Association.

[B4] Alzheimer’s Disease International (2020). *World Alzheimer Report 2020.* London, UK: Alzheimer’s Disease International.

[B5] Alzheimer’s Disease International (2021). *World Alzheimer Report 2021.* London, UK: Alzheimer’s Disease International.

[B6] Bantry W. E. Montgomery P. (2014). Dementia, walking out doors and getting lost: Incidence, risk factors and consequences from dementia-related police missing-person reports. *Aging Ment. Health* 19 224–230. 10.1080/13607863.2014.924091 24912376

[B7] Bartlett M. S. (1954). A note on multiplying factors for various chi square approximations. *J. R. Stat. Soc.* 16 296–298. 10.1111/j.2517-6161.1954.tb00174.x

[B8] Beavers A. S. Lounsbury J. W. Richards J. K. Huck S. W. Skolits G. J. Esquivel S. L. (2013). Practical considerations for using exploratory factor analysis in educational research. *Pract. Assess. Res.Eval.* 18:13.

[B9] Bessey L. J. Walaszek A. (2019). Management of behavioral and psychological symptoms of dementia. *Curr. Psychiatry Rep.* 21:66. 10.1007/s11920-019-1049-5 31264056

[B10] Brink Y. Louw Q. A. (2012). Clinical instruments: reliability and validity critical appraisal. *J. Eval. Clin. Pract.* 18 1126–1132. 10.1111/j.1365-2753.2011.01707.x 21689217

[B11] Burgio L. D. Sook P. N. Michael H. J. Sun F. (2007). A Longitudinal Examination of Agitation and Resident Characteristics in the Nursing Home. *Gerontologist* 47 642–649. 10.1093/geront/47.5.642 17989406

[B12] Cheng S. T. (2017). Dementia caregiver burden: a research update and critical analysis. *Curr. Psychiatry Rep.* 19:64. 10.1007/s11920-017-0818-2 28795386 PMC5550537

[B13] Cronbach L. J. (1951). Coefficient alpha and the internal structure of tests. *Psychometrika* 16 297–334. 10.1016/0020-7489(93)90092-9 8449658

[B14] Dudevich A. Husak L. Johnson T. Chen A. (2018). Safety and Quality of care for seniors living with dementia. *Healthc. Q.* 21 12–15. 10.12927/hcq.2018.25708 30741148

[B15] Edwards H. B. Jelena S. Whiting P. Leach V. Richards A. Cullum S. (2016). Quality of relationships as predictors of outcomes in people with dementia: a systematic review protocol. *BMJ Open* 6 1–5. 10.1136/bmjopen-2015-010835 27044583 PMC4823463

[B16] Fleming R. (2011). An environmental audit tool suitable for use in homelike facilities for people with dementia. *Australas. J. Ageing* 30 108–112. 10.1111/j.1741-6612.2010.00444.x 21923702

[B17] Gale S. A. Acar D. Daffner K. R. (2018). Dementia. *Am. J. Med.* 131 1161–1169. 29425707 10.1016/j.amjmed.2018.01.022

[B18] Goodall D. (2006). Environmental changes increase hospital safety for dementia patients. *Holist. Nurs. Pract.* 20 80–84. 10.1097/00004650-200603000-00008 16518154

[B19] Green Y. S. (2018). Safety implications for the homebound patient with dementia. *Home Health Now* 36 386–391. 10.1097/NHH.0000000000000701 30383598

[B20] Hao W. Wang Z. Zhang H. (2016). Research on the construction of home-based dementia care problem assessment sheet. *Chin. J. Nurs.* 51 1504–1508.

[B21] He Q. Wang J. Zhang Y. Wang S. Yang G. (2011). Item screening technique on clinical outcome rating scale which based on patient-reported outcomes. *Chin. J. Trad. Chin. Med. Pharm.* 26 112–114.

[B22] Kales H. C. Gitlin L. N. Lyketsos C. G. (2015). Assessment and management of behavioral and psychological symptoms of dementia. *BMJ* 350:h369. 10.1136/bmj.h369 25731881 PMC4707529

[B23] Lawshe C. H. (1975). A quantitative approach to content validity. *Personnel. Psychol.* 28 563–575. 10.1097/HMR.0000000000000243 30870220 PMC7455091

[B24] Lee L. Hillier L. M. Lu S. K. Martin S. D. Pritchard S. Janzen J. (2019). Person-centered risk assessment framework: assessing and managing risk in older adults living with dementia. *Neurodegener. Dis. Manag.* 9 47–57. 10.2217/nmt-2018-0031 30638423

[B25] Marquardt G. Bueter K. Motzek T. (2014). Impact of the design of the built environment on people with dementia: an evidence-based review. *HERD* 8 127–157. 10.1177/193758671400800111 25816188

[B26] McDermott S. (2010). Professional judgments of risk and capacity in situations of self-neglect among older people. *Ageing Soc.* 30 1055–1072.

[B27] Morse J. M. (2002). Enhancing the safety of hospitalization by reducing patient falls. *Am. J. Infect. Control.* 30 376–380. 10.1067/mic.2002.125808 12360147

[B28] National Institute on Aging (2017). *Home Safety Checklist for Alzheimer’s Disease.* Available online at: https://www.nia.nih.gov/health/home-safety-checklist-alzheimers-disease [accessed on Jan 15, 2022].

[B29] Neubauer N. A. Miguel-Cruz A. Liu L. (2021). Strategies to locate lost persons with dementia: a case study of ontario first responders. *J. Aging Res.* 2021 1–9. 10.1155/2021/5572764 34094601 PMC8140831

[B30] Newcomer R. Miller R. Clay T. Fox P. (1999). Effects of the medicare Alzheimer’s disease demonstration on medicare expenditures. *Health Care Financ. Rev.* 20 45–65. 11482124 PMC4194605

[B31] Poland F. Mapes S. Pinnock H. Katona C. Sorensen S. Fox C. (2014). Perspectives of carers on medication management in dementia: lessons from collaboratively developing a research proposal. *BMC Res. Notes* 7:463. 10.1186/1756-0500-7-463 25048052 PMC4112829

[B32] Pudelewicz A. Talarska D. Bączyk G. (2018). Burden of caregivers of patients with alzheimer’s disease. *Scand. J. Caring Sci.* 33 336–341. 30378698 10.1111/scs.12626

[B33] Smith F. Grijseels M. S. Ryan P. Tobiansky R. (2015). Assisting people with dementia with their medicines: Experiences of family carers. *Int. J. Pharm. Pract.* 23 44–51. 10.1111/ijpp.12158 25351043 PMC4369136

[B34] Somme D. Trouve H. Dramé M. Gagnon D. Couturier Y. Saint-Jean O. (2012). Analysis of case management programs for patients with dementia: a systematic review. *Alzheimers Dement.* 8 426–436. 10.1016/j.jalz.2011.06.004 22285637

[B35] UK Government Department of Health (2010). *Nothing Ventured, Nothing Gained’: Risk Guidance for People with Dementia.* London, UK: Department of Health.

[B36] Vugt M. Stevens F. Aalten P. Lousberg R. Jaspers N. Winkens I. (2004). Do caregiver management strategies influence patient behaviour in dementia? *Int. J. Geriatr. Psychiatry* 19 85–92. 10.1002/gps.1044 14716704

[B37] Waltz C. F. Strickland O. L. Lenz E. R. (2016). *Measurement in nursing and Health Research (3rd ed.).* New York, NY: Basic Books.

[B38] Wang Y. Li L. Tian S. Wu J. Wang Z. (2020). Development and psychometrics test of home environment assessment checklist for community-dwelling older adults with dementia. *J. Alzheimers Dis.* 77 1389–1396. 10.3233/JAD-200241 32925033

[B39] Wolf-Ostermann K. Meyer S. Schmidt A. Schritz A. Holle B. Wübbeler M. (2016). Users of regional dementia care networks in Germany: First results of the evaluation study Dem Net-D. *Zeitschrift Fur Gerontologie Und Geriatrie.* 50 21–27. 10.1007/s00391-015-1006-9 26779703

[B40] Woolford M. H. Weller C. Ibrahim J. E. (2017). Unexplained absences and risk of death and injury among nursing home residents: A systematic review. *J. Am. Med. Dir. Assoc.* 18 e1–e366. 10.1016/j.jamda.2017.01.007 28242190

[B41] World Health Organization (2015). *WHO Dementia Fact Sheet No. 362.* Geneva: World Health Organization.

[B42] Yin X. Tu X. Tong Y. Rui Y. Lu Z. (2012). Development and validation of a tuberculosis medication adherence scale. *PLoS One.* 7:e50328. 10.1371/journal.pone.0050328 23251363 PMC3520953

[B43] Zeng Q. J. Lu J. J. Bai G. F. Cheng Q. M. Chen C. R. (2018). Research progress on the potential safety hazards and interventions in home care of senile dementia patients. *Nurs. Integrat. Trad.Chin. Western Med.* 4 174–177.

[B44] Zhang W. Low L. F. Schwenk M. Mills N. Gwynn J. D. Clemson L. (2019). Review of gait, cognition, and fall risks with implications for fall prevention in older adults with dementia. *Dement. Geriatr. Cogn. Disord.* 48 1–13. 10.1159/000504340 31743907

[B45] Zhao H. Wang H. (2020). Care strategies for demented elderly at home during COVID-19. *Chin. J. Alzheimer’s Dis. Related Disord.* 3 161–164. 10.3389/fpsyt.2020.579842 33132939 PMC7550649

